# Associations between brain‐derived neurotrophic factor and cognitive impairment in panic disorder

**DOI:** 10.1002/brb3.1885

**Published:** 2020-10-12

**Authors:** Wenchen Wang, Yuanyuan Liu, Shuqing Luo, Xiaoyun Guo, Xingguang Luo, Yong Zhang

**Affiliations:** ^1^ Department of Bipolar Disorder Tianjin Anding Hospital Tianjin China; ^2^ Department of Cardiology Chest Hospital of Tianjin Tianjin China; ^3^ Department of Obstetrics Baoding Second Central Hospital Hebei China; ^4^ Department of psychiatry Shanghai Mental Health Center Shanghai China; ^5^ Department of Psychiatry Yale University School of Medicine New Haven CT USA

**Keywords:** brain‐derived neurotrophic factor, cognitive impairment, panic disorder, polymorphism, single nucleotide

## Abstract

**Introduction:**

Our study was designed to examine the relationship between Brain‐Derived Neurotrophic Factor (BDNF) genotypes (rs6265, Val66Met), BDNF plasma levels, and cognitive impairment in Chinese patients with panic disorder (PD).

**Methods:**

Total 85 patients with PD and 91 healthy controls finally completed all assessments. The severity of panic symptoms and whole anxiety of PD was measured by Panic Disorder Severity Scale–Chinese Version (PDSS‐CV) and Hamilton Anxiety Scale (HAMA‐14). Montreal Cognitive Assessment (MoCA) and some neurocognitive measures were conducted to evaluate the cognitive performance. All participants were detected for the plasma BDNF levels and BDNF Val66Met polymorphism before assessment and treatment.

**Results:**

No significant differences were found in the BDNF allele frequencies and the BDNF genotype distributions between healthy controls and PD patients. BDNF Met/Met genotype was associated with lower BDNF plasma levels in PD patients, and PD patients with BDNF Met/Met genotype had the lower scores in the attention and speed of processing domains compared to those with Val/Val and Met/Val genotype (*p*'s < .05). Among PD patients, the BDNF plasma levels showed moderate positive correlations with Stroop interference (*r* = .60, *p* < .001). Using the MoCA data, the BDNF plasma levels were correlated with delayed memory (*r* = .50, *p* < .001), verbal learning (*r* = .45, *p* < .001), and total scores of MoCA (*r* = .51, *p* < .001).

**Conclusions:**

The BDNF Met/Met genotype may be associated with lower BDNF plasma levels and cognitive impairments in PD patients.

## Graphical abstract

This is a study focusing on associations between Brain‐Derived Neurotrophic Factor (BDNF) single nucleotide polymorphism, plasma Levels, and cognitive impairment in Chinese patients with panic disorder (PD).We found the BDNF Met/Met genotype may be associated with lower BDNF plasma levels and some domains of cognitive impairments in PD patients.

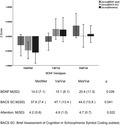

## 1 INTRODUCTION

Panic Disorder (PD) is characterized by recurrent and unexpected panic attacks and accompanied by somatic symptoms, and persistent concerns about the panic attacks (Chang et al., 2019). According to a recent study, the lifetime prevalence for PD was 1.7% in twenty‐five countries across the world. Approximately 80.4% of PD patients had lifetime comorbidity with other mental disorders, particularly with other anxiety or mood disorders (De Jonge et al., 2016). Cognitive impairment in patients with PD has attracted increasing concerns from some researches and clinical practice (Harber et al., 2019). The sustained neurocognitive impairments involving memory, attention, executive function, and speed of processing have been found in PD patients (Alves et al., 2013; O'sullivan & Newman, 2014). However, the pathophysiological mechanisms of these cognitive impairments in PD patients are still unclear.

Many previous studies have identified associations between brain‐derived neurotrophic factor (BDNF) rs6265 (Val66Met) polymorphism and depression disorders (Cardoner et al., 2013), bipolar disorders (Gonzalez‐Castro et al., 2015), and schizophrenia (Kheirollahi et al., 2016), although the findings are not always consistent (Otowa et al., 2009). In a light/dark exploration test on BDNF conditional mutant mice, the absence of central BDNF caused higher levels of anxiety and hyperactivity after exposure to stressors in these mutant mice (Rios et al., 2001). Several studies have reported that BDNF (Val66Met) single nucleotide polymorphism (SNP) could show significant impacts on episodic memory, hippocampal volume, and hippocampal neuronal function in humans (Egan et al., 2003; Han et al., 2015). Furthermore, some findings suggested that lower plasma BDNF levels might be associated with the symptomatic severity in any type of anxiety disorder, major depressive disorder, and schizophrenia (Shimizu et al., 2005; Suliman et al., 2013; Xiao et al., 2017). However, few studies have focused on the relationships between BDNF gene polymorphisms, BDNF plasma levels, and cognitive impairment in patients with PD. Therefore, we aim to investigate these relationships in Chinese PD patients to explore relevant pathophysiological mechanisms of cognitive impairment in PD.

## 2 MATERIALS AND METHODS

### 2.1 Setting and subjects

Our study was carried out in Tianjin Anding Hospital and Tianjin Chest Hospital, China. Patients (aged 16–60 years) were recruited if they: (a) had a diagnosis of PD according to the *Diagnostic and Statistical Manual of Mental Disorder, 5th ed*. (DSM‐5) criteria; and (b) had the score of the Panic Disorder Severity Scale–Chinese Version (PDSS‐CV) at least 10 and the score of the Hamilton Anxiety Scale (HAMA‐14) at least 14. Participants were excluded if they: (a) had severe physical diseases or alcohol or substance abuse; and (b) received antidepressant treatments in the past four weeks and any physical therapy such as Modified electroconvulsive therapy (MECT) and Repetitive transcranial magnetic stimulation (rTMS) in the past three months. Healthy controls (HCs) matched in education levels, gender, and age were recruited from the local community. We obtained the written informed consent from all participants, and the Ethics Institution of the two hospitals approved the study protocol.

### 2.2 Clinical symptom measures

Using PDSS‐CV and HAMA‐14 scale, the severity of panic symptoms and whole anxiety was measured by two experienced raters from two hospitals. Both raters were trained at the annual training program, the inter‐rater reliability for two raters was at a good level (the interclass correlation coefficients were higher than 0.80). The PDSS‐CV scale includes seven domains: pain caused by a panic attack, panic attack frequency, avoidance, somatesthesia, anticipatory anxiety, social functions, and occupational functions. It has been revealed to have good reliability and validity in measuring the severity of panic symptoms in Chinese patients with PD (Xiong et al., 2012). The HAMA‐14 scale comprises 14 items with good psychometric properties, including psychic anxiety and somatic anxiety domains (Wolfgang et al., 1988).

#### 2.2.1 Cognitive assessment

Montreal Cognitive Assessment (MoCA): the scale has been proved to have great psychometric properties in mild cognitive impairment patients and normal population (Chen et al., 2016). The MoCA (range: 0–30‐point) includes seven domains: visual‐spatial abilities, abstract, execution function, attention and concentration, language, delayed memory, and orientation (Nasreddine et al., 2005; Tsai et al., 2016). We also conducted other neurocognitive measures including (a) attention and speed of processing: Trail Making Test‐Part A (TMT‐A) (Klojcnik et al., 2017; Wei et al., 2018) and Brief Assessment of Cognition in Schizophrenia Symbol Coding subtest (BACS SC) (Wang et al., 2017); (b) verbal learning: Hopkins Verbal Learning Test‐Revised (HVLT‐R) (Brandt & Benedict, 2001); (c) visual memory: Brief Visuospatial Memory Test‐Revised (BVMT‐R) (Benedict, 1997); and (d) executive function: The Stroop Color‐Word Test (Klojcnik et al., 2017).

All PD patients were prescribed a fixed dose of 10 mg/day Escitalopram to treat the panic and anxiety symptoms. Besides Escitalopram monotherapy, Sedative–hypnotic drugs including zolpidem (range from 5 to10 mg/day) and zopiclone (7.5 mg/day) were allowed for night sleep within two weeks, but alprazolam and clonazepam were not permitted due to cognitive impairment.

### 2.3 Genotyping and detection of BDNF levels

We collected venous blood samples (5 ml) drawn from all participants to detect BDNF genotypes (rs6265, Val66Met) and BDNF plasma levels. The genomic DNA was extracted by using a DNA extraction and purification kit (Baio) according to the standard procedures. The BDNF rs6265 (Val66Met) SNP was performed by polymerase chain reaction (PCR) using constructed primers (forward primer: 5′‐ACT CTG GAG AGC GTG METT‐3′ and reverse primer: 5′‐ ATA CTG TCA CAC ACG CTC ‐3′).

Plasma BDNF levels (ng/ml) were measured by enzyme‐linked immunosorbent assay kits, according to the standard procedures for the kit (DG10522H, Lvyuan Biotechnology). Absorbencies at 450 nm were measured by a standard microplate reader (EL10A, BIOBASE). These samples were tested in 11 plates. The coefficients of inter‐assay and intra‐assay variations were below 15% (Wang et al., 2015). We detected BDNF plasma levels at baseline only.

### 2.4 Statistical analysis

SPSS version 25.0 was used for all data analyses. The *chi‐square* tests and independent sample *t*‐tests were used to compare the demographic information and clinical variables between PD and HC groups. For continuous variables, data are presented as means (standard deviation, *SD*). The Fisher's Exact Test was used for categorical comparisons. All BDNF SNPs in this study were tested for a *chi‐square* test‐based Hardy–Weinberg equilibrium (HWE) program. The associations across BDNF plasma levels, cognitive domains, and BDNF genotypes in PD group were assessed by one‐way ANOVA analysis. The correlation between BDNF plasma levels and cognitive performances in PD patients was calculated using partial correlation analysis. All tests are two‐tailed (*p* value < .05 is considered statistically significant).

## 3 RESULTS

### 3.1 Demographic characteristics

We recruited a total of 116 patients with PD and 99 healthy controls in our study. Thirty‐nine patients were excluded because of unwillingness to continue (22 patients), or incomplete assessments (nine patients, eight healthy controls). Total 85 PD patients and 91 healthy controls completed the final assessments. No significant differences were observed in gender, age, educational levels, and marriage status between PD and HC groups (all *p*s > .05). Table 1.

**Table 1:** Demographic characteristics between PD and HC groups

	PD (*n* = 85)	HC (*n* = 91)	*t*	*p*
Age (years)^a^	47.0 (10.8)	49.2 (10.5)	−1.37	.173
Male/Female (%)	34/51 (40.0/60.0)	39/52 (42.9/57.1)		.760^b^
Length of education (years)^a^	11.3 (3.5)	11.8 (3.01)	−1.18	.242
Unmarried/Married (*n*, %)	9/76 (10.6/89.4)	17/74 (18.7/81.3)		.143^b^
PD with/without agoraphobia (%)	24/61 (28.2/71.8)			

Abbreviations: HC, healthy control; PD, panic disorder.

^a^ Mean*(SD)*.

^b^ Statistical analysis was performed using Fisher Exact Test.

### 3.2 Clinical variables between PD group and HC group

The results illustrate that the plasma BDNF levels of PD patients were lower than that of HCs (*p* = .001). Considering gender difference, we found the plasma BDNF level of PD female patients was lower than that of female HCs (PD 15.76 ± 5.16/ HCs 21.06 ± 8.78, *p* = .003), but no significant difference of the plasma BDNF level was found between PD male subjects and HCs (*p* > .05). We observed that PD patients had lower scores in some domains of cognitive performance compared to HCs (Table 2). Besides, we also found that PD patients with agoraphobia had lower TMT‐A scores compared to those without agoraphobia (*p* = .03).

**Table 2:** Clinical variable between PD patients and healthy controls

	PD	HC	*t*	*p* value
BDNF level (ng/ml)^a^	16.9 (9.6)	21.5 (8.8)	−3.25	.001
TMT‐A^a^	48.0 (21.0)	41.5 (14.2)	2.38	.019
BACS SC^a^	43.9 (12.9)	49.0 (13.5)	−2.60	.010
HVLT‐R^a^	20.4 (5.1)	20.7 (4.2)	−0.38	.704
BVMT‐R^a^	22.0 (6.2)	23.5 (5.9)	−1.67	.096
Stroop word^a^	84.0 (14.4)	86.0 (15.8)	−0.88	.378
Stroop color^a^	59.9 (12.7)	64.5 (16.4)	−2.08	.039
Stroop interference^a^	34.2 (9.8)	40.5 (11.4)	−3.92	<.001
Total stroop^a^	178.0 (29.3)	190.9 (38.8)	−2.50	.013
Visual‐spatial abilities^a^	4.3 (0.9)	4.6 (0.6)	−2.88	.005
Abstract	1.6 (0.5)	1.9 (0.3)	−5.31	<.001
Attention and concentration^a^	4.6 (0.8)	5.0 (1.0)	−2.93	.004
Language^a^	2.5 (0.6)	2.7 (0.4)	−3.29	.001
Delayed memory^a^	2.5 (1.7)	4.2 (0.8)	−8.64	<.001
Orientation^a^	5.9 (0.2)	5.9 (0.3)	0.19	.847
Total MoCA^a^	24.3 (2.8)	27.4 (1.6)	−8.91	<.001

Abbreviations: BACS SC, Brief Assessment of Cognition in Schizophrenia Symbol Coding subtest; BVMT‐R, Brief Visuospatial Memory Test ‐Revised; HC, healthy control; HVLT‐R, Hopkins Verbal Learning Test—Revised; PD, panic disorder; TMT‐A, Trail Making Test‐Part A.

^a^ Mean (*SD*).

### 3.3 BDNF genotype and allele distributions for all subjects

The genotype distribution was corresponded with Hardy–Weinberg equilibrium for HCs (*χ* ^2^ = 3.68, *p* = .06) and PD patients (*χ* ^2^ = 3.23, *p* = .07). Using the chi‐square test, we did not find the significant differences in the BDNF genotypes and allele distributions between HCs and PD patient groups (Table 3).

**Table 3:** Genotype distribution and allele frequencies between PD patients and healthy controls

	Met/Met	Val/Val	Met/Val	*p*	Met	Val	*p*
PD (*n*,％)	18 (21.2)	34 (40.0)	33 (38.8)	.748^a^	69 (40.6)	101 (59.4)	.451^a^
HC (*n*, %)	23 (25.3)	32 (35.2)	36 (39.6)		82 (45.1)	100 (54.9)	
PD with agoraphobia (*n*, %)	8 (33.3)	8 (33.3)	8 (33.3)	.271^a^	24 (50)	24 (50)	.123^a^
PD without agoraphobia(*n*, ％)	10 (16.4)	26 (42.6)	25 (41.0)		45 (36.9)	77 (63.1)	

Abbreviations: HC, healthy control; PD, panic disorder.

^a^ Statistical analysis was performed using Fisher Exact Test.

**Figure 1:**
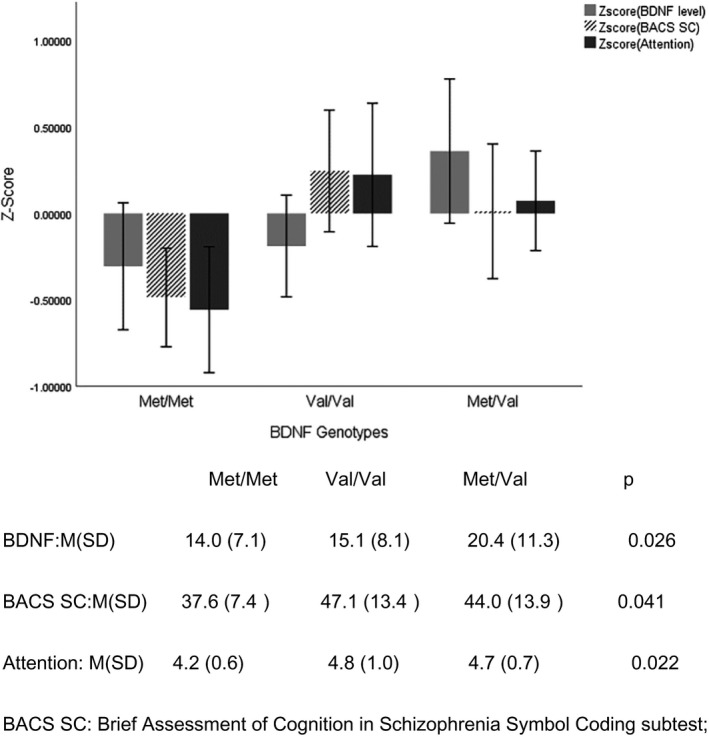
The BDNF level and cognitive performance in the three genotype distributions in PD patients. Abbreviation: BACS SC, Brief Assessment of Cognition in Schizophrenia Symbol Coding subtest

Following previous studies, based on the standard deviation and mean of each clinical variable, the raw scores of cognitive domains were standardized to *z*‐score values (Costa et al., 2015). Using one‐way ANOVA analysis, we found significant differences in the BDNF plasma levels, scores of speed of processing and attention domains between three BDNF genotypes in the PD patients (*p*s < .05). Compared to the PD patients with Val/Val and Val/Met genotype, those with Met/Met genotype had the lower scores in the afore‐mentioned domains of neurocognitive function and lower plasma BDNF levels (see Figure 1). Meanwhile, we observed that the BDNF plasma levels were positively correlated with the scores of Stroop interference (*r* = .60, *p* < .001), Stroop color‐naming (*r* = .32, *p* = .003), and total scores of Stroop test (*r* = .31, *p* = .005) even after controlling for the educational levels. Besides, using the MoCA data, the BDNF plasma levels also were positively correlated to the scores of delayed memory (*r* = .50, *p* < .001), verbal learning (*r* = .45, *p* < .001), abstract (*r* = .38, *p* < .001), and the total scores of the MoCA (*r* = .51, *p* < .001).

## 4 DISCUSSION

We preliminarily explored the relationships among BDNF gene polymorphisms, BDNF plasma levels, and cognitive impairment in Chinese PD patients. Our findings demonstrated that the lower BDNF plasma levels were associated with BDNF Met/Met genotype in PD patients, and these lower BDNF plasma levels were positively correlated with executive impairment, delayed memory, verbal learning, and abstract. Furthermore, the PD patients with Met/Met carrier could display apparent impairments of attention and speed of processing. Our findings revealed that BDNF Met/Met genotype and lower plasma levels may be susceptible to cognitive impairments in PD patients.

Consistent with previous findings in Japanese and Chinese PD patients (Konishi et al., 2014; Otowa et al., 2009), we still found no significant differences in the BDNF allele frequencies and the BDNF genotype distributions between healthy controls and PD patients.

Our findings showed that BDNF Met/Met genotype was associated with the lower BDNF plasma levels in PD patients, which is in line with an animal research (Ieraci et al., 2016). Some clinical researches also evidenced this association between the BDNF Met carrier and lower BDNF levels in the patients with major depressive disorder (Colle et al., 2017), schizophrenia, and bipolar disorder (Aas et al., 2014) respectively, indicating BDNF Met carrier may play a vital role in regulating BDNF protein expression (Youssef et al., 2018).

Our main findings revealed the PD patients with Met/Met genotype had the worse attention and speed of processing compared to those with Val/Met and Val/Val genotypes, indicating the PD patients with Met/Met genotype had difficulty to concentrate on related information and had the worse information‐processing capability. Hao et al.’s findings confirmed that BDNF Met knock‐in mice (Met/Met genotype mice) displayed poor hippocampal synaptic plasticity (Hao et al., 2017), while hippocampus and prefrontal lobe is a crucial structure involved in memory, information‐processing speed and attention (Kim et al., 2017; Rungratsameetaweemana et al., 2019). A study in healthy volunteers revealed that the BDNF Met/Met genotype had reduced gray matter volume of hippocampus than Val/Val genotype. The functional MRI suggested that during selective processing of target stimuli, the BDNF Met allele carriers were associated with more significant alterations in hippocampal‐prefrontal activation than other genotypes. These findings revealed that BDNF Met/Met genotype could present selective defects to task‐relevant information processing (Schofield et al., 2009). Moreover, Zou et al. (2019) found the BDNF Met/Met genotype is associated with the severity of anticipatory anxiety in PD patients. Some research showed that BDNF Met carriers had been associated with the decline in working memory, reduced gray matter in the hippocampus and hypofunction of the prefrontal lobe in PD patients and healthy volunteers (Gatt et al., 2009; Konishi et al., 2014). Thus we speculate that BDNF Met/Met genotype could cause these cognitive impairments by modulating the hippocampus or prefrontal function (Bird et al., 2019; Cao et al., 2016).

Unlike the previous finding (Carlino et al., 2015), our study found that the BDNF level of female PD patients was significantly lower than female HCs. Meanwhile, Molendijk et al.‘s finding revealed that female patients with anxiety disorder had lower serum BDNF levels compared to female healthy controls (Molendijk. et al., 2012). Some studies showed that females with PD have more illness burden of the health‐related quality of life, such as poor stress‐coping styles and missed work (Kim et al., 2017; Mclean et al., 2011). So we speculate that gender might mediate the association between PD and peripheral BDNF level; also, low peripheral BDNF level mignt play an essential role in the pathophysiological mechanisms of female PD patients (Molendijk. et al., 2012).

In addition, our study found moderate positive correlations between plasma BDNF levels and execution function when we used data of stroop interference test, and even showed positive correlations between plasma BDNF levels and delayed memory, verbal learning using MoCA data in PD patient. Zhu et al. (2019) found that reduced BDNF mRNA expression and protein levels could result in GABAergic neuroplasticity dysregulation and contribute to cognitive impairment. Other findings verified that the repetitive transcranial magnetic stimulation (rTMS) could enhance the synaptic plasticity (long‐term potentiation) of hippocampus region and be associated with the improvement of recognition memory deficit by increasing the level of BDNF expression in rats (Xiang et al., 2019), while decreased plasma BDNF levels were significantly correlated with delayed memory in mild neurocognitive disorder patients (Levada et al., 2016). Meanwhile, reduced serum levels of BDNF have been found in patients with panic disorder (Kobayashi et al., 2005; Strohle et al., 2010) or subtypes of anxiety disorder (Carlino et al., 2015; Molendijk. et al., 2012). A meta‐analysis also displayed that reduced BDNF levels were significantly associated with cognitive impairment in processing speed, verbal learning, and working memory in schizophrenia patients (Bora, 2019). These findings evidenced again that peripheric BDNF levels could be correlated with cognitive impairments in psychiatric disorders.

Our study had some limitations. First, we only explored the association between BDNF Val66Met SNP and cognitive impairments in PD patients; however, it is unknown whether other BDNF SNPs such as rs11030104 and rs7103411 could be involved in BNDF plasma Levels and cognitive defects in PD patients (Han et al., 2015). We should pay attention to other BDNF SNPs and their correlations with BDNF plasma levels and cognitive performance, although no relevant researches were yet found in PD patients. Second, lack of endpoint BDNF level measurements may weaken the correlation between cognitive function and improvement in BDNF levels. Third, it is hard to convincingly elucidate the mechanism underlying the cognitive deficits due to lack of brain imaging or electrophysiologic resources. Last, insufficient sample size inevitably cripples statistical power when we analyze the association between BDNF genotypes and cognitive domains as well as BDNF plasma levels.

In summary, our findings also implied that PD patients with BDNF Met/Met genotype could be the target population when we carry out early cognitive remediation. Further research should examine the possible biological correlation between cognitive performances and multiple neurotrophic factors in Chinese PD patients.

## CONFLICT OF INTEREST

All the authors stated no conflict of interest.

## AUTHORS’ CONTRIBUTIONS

Xingguang Luo, Yong Zhang contributed to the conception of the study, Shuqing Luo, Xiaoyun Guo contributed significantly to analysis and manuscript preparation; Wenchen Wang, Yuanyuan Liu performed the data analyses and wrote the manuscript. All the authors approved the final version of the article.

## ETHICAL APPROVAL

The local ethics committee is the Human Ethics Review boards of Tianjin Anding Hospital and Tianjin Chest Hospital. This study was performed in accordance with the tenets of the Declaration of Helsinki. All participants provided their written informed consent to investigators after a complete study explanation.

### Peer Review

The peer review history for this article is available at https://publons.com/publon/10.1002/brb3.1885.

## Data Availability

The data that support the findings of this study are available from the corresponding author upon reasonable request.
